# Multifactorial Evaluation of Regrouping Effects on Performance and Welfare in Two Italian Dual-Purpose Chicken Breeds: *Bianca di Saluzzo* and *Bionda Piemontese*

**DOI:** 10.3390/ani12182355

**Published:** 2022-09-09

**Authors:** Sihem Dabbou, Cecilia Mugnai, Dominga Soglia, Martina Tarantola, Elisabetta Macchi, Stefano Sartore, Stefania Bergagna, Giulia Pagliasso, Achille Schiavone

**Affiliations:** 1 Center Agriculture Food Environment (C3A), University of Trento, Via E. Mach 1, 38010 San Michele all’Adige, Italy; 2 Research and Innovation Centre, Fondazione Edmund Mach, 38010 San Michele all’Adige, Italy; 3 Department of Veterinary Science, University of Turin, Largo P. Braccini 2, 10095 Grugliasco, Italy; 4 Veterinary Medical Research Institute for Piemonte, Liguria and Valle d’Aosta, Via Bologna 148, 10154 Turin, Italy

**Keywords:** *Bionda Piemontese*, slow-growing chicken, stressful housing practice, welfare, performance

## Abstract

**Simple Summary:**

Local chicken breeds *Bionda Piemontese* (BP) and *Bianca di Saluzzo* (BS) are promising for use in small-scale poultry farms, which adopt free-range and/or organic farming practice. However, in practical conditions, it can happen that animals are subjected to regrouping, creating a new flock by mixing different groups. Such situations may provoke a stressful response in birds, compromising their welfare. To evaluate the resilience of BP and BS breeds to adverse management practices (regrouping), the present study aimed to assess the birds’ welfare status through a multifactorial approach. As a result, the practice of regrouping immediately compromised the welfare status and the productive performances in both BP and BS hens, but, in the following weeks, birds displayed a reliance, which help them adapt to the new stressful conditions.

**Abstract:**

The aim of the present study was to investigate the ability of two Italian slow-growing poultry breeds (namely, *Bionda Piemontese*, BP, and *Bianca di Saluzzo*, BS) to cope with a stressful event, such as collective grouping, using a multifactorial approach. A total of 120 hens of BP and BS breeds were homogenously distributed, according to breed, in 12 pens (10 hens/pen; 6 pens/breed), from 18 to 49 weeks of age. At 50 weeks of age, hens were regrouped (Stressful Farm Practice, SFP), by removing separators, both in indoor and outdoor areas. At 49 weeks of age, 24 hens/breed were randomly selected for the evaluation of welfare (ethological and physiological) parameters, at different time points: T0: 1-week pre-SPF; T1: 1-week post-SFP; T2: 3-week post-SFP; T3: 5-week post-SFP. Egg production was recorded from 38 to 56 weeks of age. Grouping produced a social stress in both BS and BP-laying hens, which was expressed in terms of productive traits (reduction of oviposition), behavioral modification (worsening of plumage condition due to feather peaking and extension of the duration of the tonic immobility test) and physiological modification (increased heterophil/lymphocyte ratio and corticosterone metabolites in droppings). Both breeds reacted in a similar way; in fact, no differences were attributed to the breed. At the end of the observation period, the egg rate fully recovered, while the behavioral and physiological parameters partially recovered but failed to recover to those recorded prior to the stressful event.

## 1. Introduction

In the last few years, consumers are showing a rising interest in the organic and free-range chicken meat and eggs, which has consequently led to increased production [1]. However, this demand also regards animal welfare, environmental impact, and qualitative characteristics of the farming system from which these products originate. Alternative poultry farming promotes biodiversity preservation, free-range farming system, local economies, and farm multi-functionality. Furthermore, alternative poultry farming provides eggs and meat to which consumers attribute high ethical value, quality, and taste, despite its high cost in comparison with products from the intensive system [2]. In developed countries, the safeguard and preservation of poultry biodiversity is a challenge for sustainable production of food and land management in the era of climate change [3]. Stress in poultry may produce adverse effect on the health status and productivity. The EC regulations and the final recommendation of the Network for Animal Health and Welfare in Organic Agriculture suggest to improve the farming of slow-growing breeds [4,5], characterized by a high tendency to pasture, adaptability to poor environment (high foraging aptitude, immune response, and thermo-tolerance), and climatic variations [6,7,8].

Despite a technical limitation and funding support to the small-scale farmers, local chicken breeds are protected as part of various conservation programs in various Italian regions [9,10]. *Bionda Piemontese* (BP) and *Bianca di Saluzzo* (BS) breeds are the most widespread local chicken breeds of the Piedmont region (Northwest Italy), mainly reared for meat and egg production. Both breeds have been recently classified as slow-growing chickens [11] and relevant research has been carried out concerning the preservation of biodiversity, genetic and performance improvement of both BP and BS breeds [12,13,14,15,16,17,18].

In Italian small-scale farming, a common practice is regrouping of different groups of birds, modifying their rearing areas (indoor and/or outdoor) to meet farm organization requirement [9,10], without taking account of stress effects on welfare and performance of birds. Thus, in this context, multifactorial stress studies would be a crucial key to improve the technical support to local poultry farmers’ development, thus contributing to safeguard poultry welfare. Stress in poultry has adverse effect on the health and the productivity of the birds, it reduces the efficiency of the immune system and the bird’s productivity. Adaptability to a new housing system involves multiple aspects, such as physiological status, well-being, and behavioral traits, requiring the analysis of numerous parameters that can cause stress [7,19,20]. Haemisch and Gartner [19] pointed out that social stress reaction is a strain-specific interaction between dominance hierarchies and environmental effects. Housing environment or regrouping individuals from the different strains or groups can cause social stress [20]. Little attention has been given to autochthonous breeds’ response to stress conditions, such as density, type of rearing system, change of environment in respect of genotype, and transport [20,21,22,23,24,25,26]. Therefore, the aim of the present study was to investigate the resilience to regrouping in two Italian poultry breeds (BP and BS), on birds’ welfare and performance, through a multifactorial approach, which included the monitoring of productive, ethological, and physiological traits.

## 2. Materials and Methods

### 2.1. Ethical Statement

The experimental protocol was approved by the Bioethical Committee of the University of Turin (prot. no. 451944). Blood samples were collected during routine health controls and the farm was considered a sentinel farm for surveillance of salmonella and avian flu by the Public Veterinary service. No action involving pain or suffering was practiced.

### 2.2. Birds Housing Condition and Diet

The trial was performed at the Giuliano Giuseppe family farm (Str. Vicinale di Bruino, 5-10040 Volvera, Torino, Italy) (44.96272546630037, 7.509775655821441). Sixty pullets of *Bianca di Saluzzo* (BS) and of *Bionda Piemontese* (BP) were housed at the age of 18 weeks in a poultry house with 12 indoor pens (2.2 × 3.5 m), each with access to an external paddock (2.2 × 4.5 m). Each pen housed 10 birds and 6 pens were assigned to each breed.

At the age of 50 weeks, for each breed, the dividing walls between the six pens were removed, in order to create a single pen (stressful farming practice, SFP), both in the indoor and outdoor areas. The density remained the same and the final dimensions for each pen per breed were 13.2 × 3.5 m for the indoor area and 13.2 × 4.5 m for the outdoor area. Throughout the trial, a natural photoperiod was applied. The birds always had free access to water and were fed ad libitum a standard commercial diet (165 g/kg crude protein and 11.20 MJ/kg apparent metabolizable energy). Among all the hens raised, 4 hens/pen/breed were randomly chosen (24 hens per breed in total) and individually identified with a wing mark at the age of 49 weeks, one week before the regrouping. The same birds underwent the welfare multifactorial evaluation (behavioral, hematological, and hormonal) as described in the following sections. The sampling time was settled as follows: T0 at 49 weeks of age (1-week pre-SFP), T1 at one week after the SFP (51 weeks old), T2 at 3-week post-SFP (53 weeks old); and T3 at 5-week post-SPF (55 weeks old). Morbidity and mortality were also daily monitored.

### 2.3. Productive Performance

The egg production of all the hens was daily recorded from 38 to 56 weeks of age.

The daily egg-laying rate was calculated by dividing the number of eggs by the number of live hens in both breeds and the weekly laying rate was averaged. With the values, a graph was obtained to observe the changes in the trend of egg production before and during the study period.

### 2.4. Tonic Immobility and Plumage Evaluation

The selected hens were tested at T0, T1, T2, and T3 for tonic immobility test (*TI)* in a separate room [26]. Each individual was carefully and gently caught with both hands, held in an inverted manner, and carried to a separate room (nonvisual contact with other hens) for the *TI* measurements. *TI* was induced by placing the bird on its back with its head hanging in a U-shaped plastic cradle and gently restraining the bird on the breastbone for 10 s. The observer then retreated approximately 1 m, avoiding direct eye contact with the bird because of the fear-inducing properties of eye contact, and limiting unnecessary noise or movement. A stopwatch was started to record latency until the hen righted itself. If the bird righted itself in less than 10 s, the restraining procedure was repeated. If *TI* was not induced after three attempts, the duration of *TI* was considered 0 s. If the bird did not show a righting response over the 3-min test period, a maximum score of 180 s was given for the righting time. Thus, tonic immobility duration ranged from 0 to 180 s.

Immediately after the *TI* test, the plumage condition evaluation was assessed following a 4-point scale for each trait, where a score of 4 implied the best condition and a score of 1 the worst [27]. The observation focused on six parts of the body: neck, breast, belly, wings, back, and tail, implying a total score ranging from 6 to 24 points.

Individual scores of <2 indicate severe damage of the plumage due to pecking or wear. In particular, aggressive pecks in the neck area, bad conditions of the tail and rear plumage indicate the presence of the pecking phenomenon. The sum of the score of each area of the body offers a general overview of the conditions of the plumage of chickens. A total score lower than 10–12 indicates severe damage to the plumage on the whole body or on a large part of the body. A single score of >3 (for each of the six considered areas) and a total score of >18–20 would indicate a good feather cover [19].

### 2.5. Hematological Parameters and Droppings’ Corticosterone Metabolites Concentration

At same time points, after *TI* and plumage evaluation, blood samples were collected from the ulnar vein for the measurement of blood stress parameters. A total of 2.5 mL was placed in a serum-separating tube. Then, the blood smear was prepared, using one glass slide for each bird, from a drop of blood without anticoagulant. The smears were stained using May–Grünwald and Giemsa stains [28]. The total erythrocytes and leucocytes cell counts were determined in an improved Neubauer hemocytometer (Qiujong^®^, Benjing, China) on blood samples previously treated with 1:200 Natt–Herrick solution (Bioanalytic GmbH, Freiburg im Breisgau, Germany). A total of 100 leukocytes, including granular (heterophils, eosinophils, and basophils) and non-granular (lymphocytes and monocytes) leucocytes, were counted on the slide, and the heterophilic ratio on lymphocytes (H/L) was also calculated [29,30].

A droppings sample (n = 24) was taken from each hen subject to the study, immediately after defecation. For this purpose, after the *TI* test and plumage evaluation, each hen was housed in wire mesh cages (100 cm × 50 cm width × length) for approximately 10 minutes to collect fresh excreta samples, then the birds were released in the pen. The excreta of each animal were stored at −20 °C until lyophilization (Edwards MF 1000, Milan, Italy) and analyses. To extract the corticosterone metabolites (CMs), we used the methanol-based procedure described by Palm et al. [31] with slight modifications reported by Costa et al. [32]. An aliquot of the samples (0.25 g each) was placed into extraction tubes, which were then sealed with a Teflon cap and stored at −20 °C. Each aliquot was thoroughly mixed for 20 min using a multivortex with 3 mL of 80% methanol (Sigma Aldrich, St. Louis, MO, USA). The solid part on the bottom was left to settle and the test tube was placed in the freezer for two hours to freeze the sediment on the bottom. After 2 h, an aliquot (0.5 mL) of the supernatant was transferred into a new vial and evaporated for 14 h. After evaporation, the dried extracts were stored in dark boxes in the freezer for 7 days before measurement. For the measurement of CMs, a multispecies ELISA kit (K014; Arbor Assays^®^, Ann Arbor, MI, USA) was validated for different matrices: serum, plasma, urine, dehydrated faeces, and tissue cultures were used. An aliquot of the extract was diluted to 1:10 in the assay buffer. The mixture was then vortexed and left to rest for 5 min and repeated a second time to ensure complete steroid solubility. All of the analyses were repeated twice. The inter- and intra-assay coefficients of variation were less than 10% (7% and 9%, respectively). The sensitivity of the assay was 11.2 ng/g droppings. All of the dropping samples were analyzed at multiple dilutions (1:4, 1:8, 1:16 and 1:32), and all regression slopes were parallel to the standard curve (r^2^ = 0.987). The mean recovery rate of corticosterone added to dried excreta was 96.4%. According to the manufacturer, the corticosterone kit (K014; Arbor Assays^®^, Ann Arbor, MI, USA) presents the following cross reactivity: 100% with corticosterone, 12.3% with desoxycorticosterone, 0.62% with aldosterone, 0.38% with cortisol, and 0.24% with progesterone. The concentration of CMs was expressed as ng/g of droppings dry matter (DM).

### 2.6. Statistical Analysis

The statistical analysis was performed using the SPSS software package (version 21 for Windows, SPSS Inc., Chicago, IL, USA). Before testing for group differences, the normality of the data distribution and the homogeneity of variance were assessed using the Shapiro–Wilk test and Levene’s test, respectively. To carry out the analysis on the level of oviposition between the two breeds, the Chi-square test (χ^2^) was performed on their oviposition curves, both on the data collected weekly and on those of the whole period. The effect of SFP on all studied parameters in the two breeds across four sampling time (T0, T1, T2 and T3) was statistically analyzed with mixed between-within subject models (GLM-repeated measures). Significance was declared at *p* < 0.05. A statistical trend was considered at *p* < 0.1.

## 3. Results

### 3.1. Productive Performance

No morbidity and mortality were recorded during the experimental periods.

**Figure 1:**
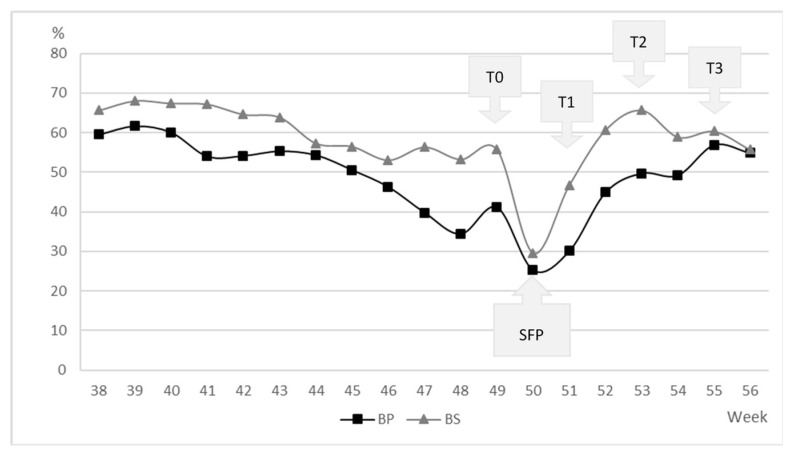
Egg-laying rate (%) of *Bianca di Saluzzo* (BS) and *Bionda Piemontese* (BP) laying hens from 38 to 56 weeks of age. T0: 1-week pre-Stressful Farming Practice (SPF); T1: 1-week post-SFP; T2: 3-week post-SFP; T3: 5-week post-SFP.

The egg-laying rate, from 38 to 56 weeks of age, of both BP and BS is presented in Figure 1. The breed-affected egg production rate at different experimental times (from 38 to 56 weeks, *p* < 0.01), with the BS breed having a higher egg production rate. Before SFP (from 44 to 49 weeks of age), the average percentage of egg production was 50.94 ± 8.72 and 60.78 ± 5.85, for BP and BS, respectively (*p* < 0.01). The oviposition peak was observed at 39 weeks of age, corresponding to 69.32% for BS and 60.97% for BP, respectively (*p* < 0.05).

In both breeds, a decrease in the egg production was recorded at SFP (25.26% and 29.64% for BP and BS, respectively; *p* > 0.05). Whereas, in both breeds, after SFP, from 51 to 55 weeks of age, the egg-laying rate increased progressively to an average of 30.04% and 47.62%; 50.03% and 64.60%; 57.32%, and 60.20%, for BP and BS at T1, T2, and T3, respectively (*p* < 0.01).

### 3.2. Tonic Immobility Test and Plumage Conditions

The data on TI test are reported in Table 1. No significant difference was observed in relation to the breed and the interaction time × breed. Interestingly, a statistical trend was observed in relation to the effect of sampling time (*p* = 0.055) over the TI duration. The TI duration was +67.43%, +57.61%, and +43.16% longer than T0 (103.6 s) at T1, T2, and T3, respectively.

The plumage conditions in all the body areas of the two breeds were scored around 4 at T0 (plumage not damaged) (total score 23.65/24.00). The sampling time had a significant effect (*p* < 0.05) on plumage conditions of belly, neck, tail, wings, and tail body parts. Belly was the only body part that showed a significant effect for breed, time, and their interaction (*p* < 0.05). No effect of sampling time was observed for breast and back plumage conditions. The total plumage score was −1.48%, −13.82%, and −9.98% lower than T0 at T1, T2, and T3, respectively.

### 3.3. Hematological Parameters and Droppings’ Corticosterone Metabolites (CMs) Concentration

The hematological parameters and CM concentration of the BP and BS hens are reported in Table 2. A significant effect of sampling time on the leucocytes, H/L ratio, and corticosterone metabolites values was observed (*p* < 0.05). No significant differences were observed between the two breeds. No significant interaction between breed and sampling period was reported for any of the studied variables. Erythrocytes remained stable over time while leucocytes values slightly decreased from T0 to T1 and, subsequently, increased progressively up to T3 (35 days post-SFP). The leucocytes’ concentration at T3 was 18.32% higher than at T0. Both H/L ratio and CMs displayed a similar pattern over time, with sampling time having a significant effect on both parameters (*p* < 0.05). The lowest value of both H/L ratio and CMs was observed at T0, then both values increased at T1, reaching a peak at T2, and slightly decreased at T3. At T2, the H/L ratio and CMs values were +22.5% and +67.17% greater than at T0, respectively.

## 4. Discussion

Native breeds are mainly used for organic or free-range farming system in a rural context. In these practical farming conditions, especially in small-scale poultry farms, regrouping may occur. Thus, our challenge was to evaluate the resilience of two native laying hens’ populations, namely BP and BS, to SPF condition, by monitoring both productive and welfare parameters.

In the present study no morbidity or mortality were observed over time.

There was a drop in the egg production immediately after the SFP. This highlights the stressful condition induced by the new environment and social pattern of the new flock. Several studies confirmed that stressful agents cause a reduction in reproductive performance in laying hens [33,34]. At T2 and T3, in both breeds, the egg production gradually increased, probably as birds adapted to the new grouping, with the BS egg production being greater than that of the BP birds, as during the previous weeks. At T2, the egg production rate achieved by the BS breed was close to the oviposition peak, suggesting a greater resilience of BS to SPF than the BP breed.

Concerning the welfare parameters, the results of the current study showed that regrouping had a significant effect both on ethological (*TI* and plumage score) and physiological (blood traits and CMs) parameters.

The *TI* test is a tool used to evaluate fearfulness in birds [35,36,37] and represents a defensive reaction that may be used to measure the wellbeing and/or stress levels in poultry [36]. The statistical trend, which was close to significant (*p* = 0.055), increased the duration of *TI* over time, suggesting that SFP was a stressful situation for the hens. In fact, the minimum of *TI* was observed at T0, 1 week before the SFP, and the peak at T1, 1 week after SFP, followed by a progressive decrease at T2 and T3. However, at T3, the *TI* duration remained +43.16% higher than at T0, suggesting that after a stressful event such as regrouping, five weeks were not sufficient time to fully recover the previous ethological pattern. The lack of differences between the two breeds concerning *TI* duration may be attributed to a similar genetic response to stress in these slow-growing dual-purpose breeds [25,37,38,39,40]. Similarly, Ferrante et al. [25] did not observe any difference in *TI* duration among BP and two other Italian slow-growing chicken breeds (namely, Valdarnese Bianca and Robusta Maculata).

Enhanced feather pecking and aggressive behaviors could result from stress adaptation [41,42]. Accordingly, in relation to plumage score, the results of our study highlighted the worsening of general plumage conditions due to presence of feather pecking in both breeds. The worst result of the plumage condition was observed at T2 (3-week post-SFP), because of the new social interaction occurring after the regrouping. In fact, the body areas that showed worst condition were the belly, the neck, the tail, and the wings, which are the areas that birds can easily peck. Among those body areas, the parts most affected by feather pecking were tail and wings, which correspond to the areas frequently affected by non-aggressive feather pecking behaviors characterized by gentle pecks [43]. On the contrary, breast and back were not subjected to significant feather pecking and its plumage score was unaffected over sampling time. Furthermore, our results agree with those presented by Patzke et al. [44], who found a correlation between social stress and the living environment of laying hens. Previous studies reported that the increased flock size is associated with reduced birds’ feather pecking score [44,45,46]. In the present study, a slight improvement of whole plumage score was observed at T3. Therefore, considering the time necessary for feather regrowth, which is approximatively 3–6 weeks [47], it can be assumed that the enhanced feather pecking behavior associated with establishing a new social hierarchy occurred only between T1 and T2. The breed did not influence the feather score, suggesting that both genotypes reacted similarly to the regrouping.

In our study, the leukocyte counts decreased after the SPF and increased in the following weeks at T2 and T3. An increase in total leucocytes’ counts have been reported in animal models exposed to stressors [48,49,50,51,52,53,54,55] because of noradrenaline production [50]. Furthermore, previous studies reported that psychological stress has been shown to influence the number and distribution of leukocytes in the blood in a rapid and reversible manner [49,50,51,52,53].

It is well known that in avian species, the H/L ratio increases under different stressors [40,49,51] related to stocking density, housing, environmental conditions, and management practices [51,52,53,54]. Furthermore, some authors reported that there is a genetic component of the H/L ratio response to stressors [54,55,56,57,58]. The augmentation of the H/L ratio is an indicator for stressful conditions or infections in animal environments, and it is often associated with an increase in the corticosterone concentrations [48,50,54,55]. In our study, the H/L ratio was always higher after SPF than at T0, with a peak reached at T2.

The concentrations of corticosterone in droppings have been used in poultry as an indicator of stress response [59,60]. In our study, the droppings’ CMs increased over time after regrouping, confirming that a social stress occurred. The CMs concentration reached a peak at T2 and, then, it slightly decreased at T3; however, without reaching the basal level observed at T0. Surprisingly, this pattern is the same as that observed for the H/L ratio. On the other hand, both the concentration of droppings’ corticosterone and the H/L ratio did not show any difference between the two breeds. The genetic background may influence the bird’s response to stressors; in fact, Ferrante et al. [25] showed differences in plasma corticosterone and H/L ratio between three Italian autochthonous breeds with lower values in the BP breed compared to other slow-growing breeds. However, in the study of Ferrante et al. [25], the BS breed was not included, so a direct comparison is not possible.

## 5. Conclusions

Regrouping produced a social stress in both BS and BP laying hens, which was expressed in terms of productive traits (reduction of oviposition), behavioral modification (worsening of plumage condition due to feather peaking and extension of the duration of the tonic immobility test), and physiological modification (increased H/L ratio and droppings corticosterone metabolites concentration). Both breeds react in a similar way, in fact, no differences were attributed to the breed, probably associated to genetic similarity due to their common geographical origin. Interestingly, all the parameters considered in this multifactorial evaluation displayed a coherent trend with a worsening of all the observed parameters immediately after the stressful event, followed by a progressive recovery. This pattern suggests two hypotheses: the experimental period was not long enough to allow the observation of a complete recovery of the behavioral and physiological parameters to the basal level, or the new flock size provokes complex social interactions, which may continuously stimulate the hypothalamic–pituitary–adrenal axis and thus the stress response.

## Figures and Tables

**Table 1:** Effect of regrouping on tonic immobility test (TI) and plumage conditions in *Bianca di Saluzzo* (BS) and *Bionda Piemontese* (BP) laying hens according breed and sampling time (n = 24).

	Breed (B)			Sampling Time (T)							
Parameters	BS	BP	RMSE	T0	T1	T2	T3	RMSE	B	T	T × B
*Tonic immobility test*											
*TI*	151.3	143.5	25.887	103.8	173.8	163.6	148.6	51.77	0.512	0.055	0.426
*Plumage Condition*											
Breast	4.00	4.00	0.00	4.00	4.00	4.00	4.00	0.00	n.s	n.s	n.s
Belly	3.63	4.00	0.28	4.00 ^A^	4.00 ^A^	3.63 ^B^	3.63 ^B^	0.56	0.008	0.008	0.008
Neck	3.70	3.80	0.34	4.00 ^A^	4.00 ^A^	3.50 ^B^	3.50 ^B^	0.68	0.518	0.004	0.518
Tail	3.43	3.58	0.41	4.00 ^A^	4.00 ^A^	2.75 ^B^	3.25 ^B^	0.81	0.421	<0.001	0.419
Wings	3.49	3.55	0.48	4.00 ^A^	4.00 ^A^	2.85 ^B^	3.23 ^B^	0.95	0.773	0.001	0.930
Back	2.65	2.34	1.10	3.65	3.60	3.65	3.68	0.86	0.533	0.467	0.827
Total	20.92	21.27	0.96	23.65 ^a^	23.30 ^a^	20.38 ^b^	21.29 ^b^	0.63	0.093	0.032	0.098

T0: 1-week pre-Stressful Farming Practice (SPF); T1: 1-week post-SFP; T2: 3-week post-SFP; T3: 5-week post-SFP; H/L: Heterophil to Lymphocyte ratio; RMSE: Root Mean Square Error. ^A,B^: *p* < 0.01; ^a,b^: *p* < 0.05.

**Table 2:** Effect of regrouping on hematological parameters and droppings corticosterone metabolites (CMs) concentrations in *Bianca di Saluzzo* (BS) and *Bionda Piemontese* (BP) laying hens according breed and sampling time (n = 24).

	Breed (B)			Sampling Time (T)					*p* Values		
	BS	BP	RMSE	T0	T1	T2	T3	RMSE	B	T	T × B
Erythrocytes (10^6^ cell/µL)	3.91	4.10	4.47	3.67	3.65	3.72	3.68	1.83	0.341	0.191	0.595
Leucocytes (10^3^ cell/µL)	1.51	1.48	1.41	1.45 ^B^	1.36 ^C^	1.44 ^B^	1.72 ^A^	2.83	0.643	0.005	0.342
H/L ratio (%)	0.92	0.87	0.17	0.80 ^c^	0.88 ^b^	0.98 ^a^	0.92 ^a^	0.14	0.548	0.022	0.467
CMs (ng/g DM)	69.91	63.16	9.28	47.22 ^C^	66.29 ^B^	78.94 ^A^	73.68 ^A^	18.57	0.121	<0.001	0.306

T0: 1-week pre-Stressful Farming Practice (SPF); T1: 1-week post-SFP; T2: 3-week post-SFP; T3: 5-week post-SFP; H/L: Heterophil to Lymphocyte ratio; RMSE: Root Mean Square Error. ^A,B,C^: *p* < 0.01; ^a,b,C^: *p* < 0.05.

## Data Availability

Not applicable.
